# Influenza activity prediction using meteorological factors in a warm temperate to subtropical transitional zone, Eastern China

**DOI:** 10.1017/S0950268819002140

**Published:** 2019-12-20

**Authors:** Wendong Liu, Qigang Dai, Jing Bao, Wenqi Shen, Ying Wu, Yingying Shi, Ke Xu, Jianli Hu, Changjun Bao, Xiang Huo

**Affiliations:** 1 Jiangsu Provincial Center for Disease Control and Prevention, Nanjing, China; 2 Jiangsu Meteorological Service Center, Nanjing, China

**Keywords:** Forecast, influenza activity, meteorological factor, random forest model

## Abstract

Influenza activity is subject to environmental factors. Accurate forecasting of influenza epidemics would permit timely and effective implementation of public health interventions, but it remains challenging. In this study, we aimed to develop random forest (RF) regression models including meterological factors to predict seasonal influenza activity in Jiangsu provine, China. Coefficient of determination (R^2^) and mean absolute percentage error (MAPE) were employed to evaluate the models' performance. Three RF models with optimum parameters were constructed to predict influenza like illness (ILI) activity, influenza A and B (Flu-A and Flu-B) positive rates in Jiangsu. The models for Flu-B and ILI presented excellent performance with MAPEs <10%. The predicted values of the Flu-A model also matched the real trend very well, although its MAPE reached to 19.49% in the test set. The lagged dependent variables were vital predictors in each model. Seasonality was more pronounced in the models for ILI and Flu-A. The modification effects of the meteorological factors and their lagged terms on the prediction accuracy differed across the three models, while temperature always played an important role. Notably, atmospheric pressure made a major contribution to ILI and Flu-B forecasting. In brief, RF models performed well in influenza activity prediction. Impacts of meteorological factors on the predictive models for influenza activity are type-specific.

## Introduction

Seasonal influenza has always been a major public health problem [1, 2]. It annually causes tens of millions of respiratory illnesses and hundreds of thousand deaths worldwide [3]. An accurate forecast of influenza activity in advance based on predictive models is crucial for public health authorities to predict the seasonal fluctuation and facilitate key response actions [4, 5], such as public health surveillance, deployment of emergency supplies and hospital resource management. However, accurate prediction remains a great challenge. A number of statistical approaches have been employed and evaluated. Random forest (RF) regression model was suggested to have enhanced prediction ability over the autoregressive integrated moving average (ARIMA), the generalized linear autoregressive moving average time series model [6, 7] in context of animal influenza activity prediction. It performed better in identifying independent factors associated with H1N1pdm influenza infections over boosted regression trees, conventional and penalised logistic regression [8].

Meteorology plays an important role in the varied seasaonal patterns of influenza in temperate, subtroptical and tropical regions. Influenza activity has been reported to peak during rainy seasons in tropical climates and during dry, cold months of winter in temperate climates. The impact of climate conditions on influenza A and B could be different [9].

Influenza like illness (ILI) has been commonly used as the index of influenza activity worldwide [3–5], however, a number of respiratory pathogenicities, including parainfluenza, adenovirus and rhinovirus, could cause ILI and thus, influence ILI activity fluctuation [10]. Recently, the positive rate of influenza virus in surveillance samples has been considered a more reliable indicator of influenza activity [11,12].

Jiangsu Province is situated in the middle east coast of China and is a transitional district of warm temperate zone to subtropical zone. Researches conducted in this region could deliver a more comprehensive understanding of climate impact on influenza activity. In this study, we aim to develop RF models to predict the ILI activity, positive rates of Flu-A and Flu-B, respectively, which has been rare in published studies.

## Materials and methods

### Data sources

Surveillance of ILI and influenza virus in China is conducted through a national sentinel network [13], with sentinel sites covering 2.5% of all hospitals across the country. Data for patients fitting the definition of ILI (i.e. body temperature ⩾38 °C with a cough and/or a sore throat) is reported to the China Influenza Surveillance Information System (CISIS) on a weekly basis. In Jiangsu, for each sentinel site, no less than 20 nasopharyngeal swabs are collected in a week by convenience samples of ILI cases before antiviral therapy. These specimens are routinely tested for influenza virus subtypes using real-time fluorescent quantitative polymerase chain reaction (PCR) assay and the results are reported to CISIS within 48 h.

In this study, weekly data of ILI percentage in outpatients (ILI%) and influenza virus positive rate in Jiangsu during 2011–2016 were obtained from CISIS. The daily meteorological data were downloaded from China Meteorological Data Sharing Service System (http://cdc.cma.gov.cn) and aggregated into weekly data. These meteorological viriables include precipitation (PR), sunshine duration (SD), relative humidity (RH), atmospheric pressure (AP), minimum temperature (MIN_T), mean temperature (MEAN_T) and maximum temperature (MAX_T).

### Random forest

Rf is an ensemble machine learning method proposed by Breiman [14]. RF creates multiple classification and regression trees, each trained on a bootstrap sample of the original training data with a randomly selected subset of input variables. There are two parameters to choose when running a RF algorithm: the number of trees and the number of randomly selected variables. In regression, the tree predictor takes on numerical values as opposed to class labels used by the RF classifier. RF regression models take the average of outputs produced by the trees as the final prediction.

One of the most important features of RF is to calculate the variable's importance, which measures the association between a given variable and the prediction accuracy. RF regression approach discussed in this study uses the decrease in accuracy to assess the variable's importance. As suggested by previous studies about the good prediction capacity, we explored RF method in human seasonal influenza activity analyses, testing its forecasting and independent influence factors identifying performance.

### Model evaluation

Data of 2011–2015 were split as a training set to fit the RF models, reserving 2016 as testing set to evaluate the predicting accuracy. Coefficient of determination (R^2^) and mean absolute percentage error (MAPE) were employed to evaluate the models' performance both in the model fitting stage and prospective forecasting stage. They were calculated as follows:
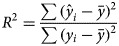

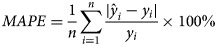
where *y*_*i*_ means the *i*^th^ observation, 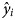 means the *i*^th^ predication, 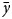 means average of observations and *n* is the number of observations.

### Statistics analysis

Descriptive statistics was used to illustrate the temporal pattern of ILI% and the influenza virus positive rate. Time series analysis methods were employed to identify the autoregressive order [15] of the dependent variables (i.e. ILI%, positive rate of Flu A and positive rate of Flu B). Cross correlation is a measure of association of a time series with another time series at different lags [16], which is essentially a univariate correlation method. In this study, cross correlation was used to determine the lag of climate variable that was most significantly associated with dependent variables. All the analyses in this study were completed using R version 3.5.0. Particularly, cross-correlation analyses were completed using the R package ‘TSA’. RF model fitting and forecasting were done in the R package‘randomForest’ [17].

## Results

### General description

**Fig. 1.**
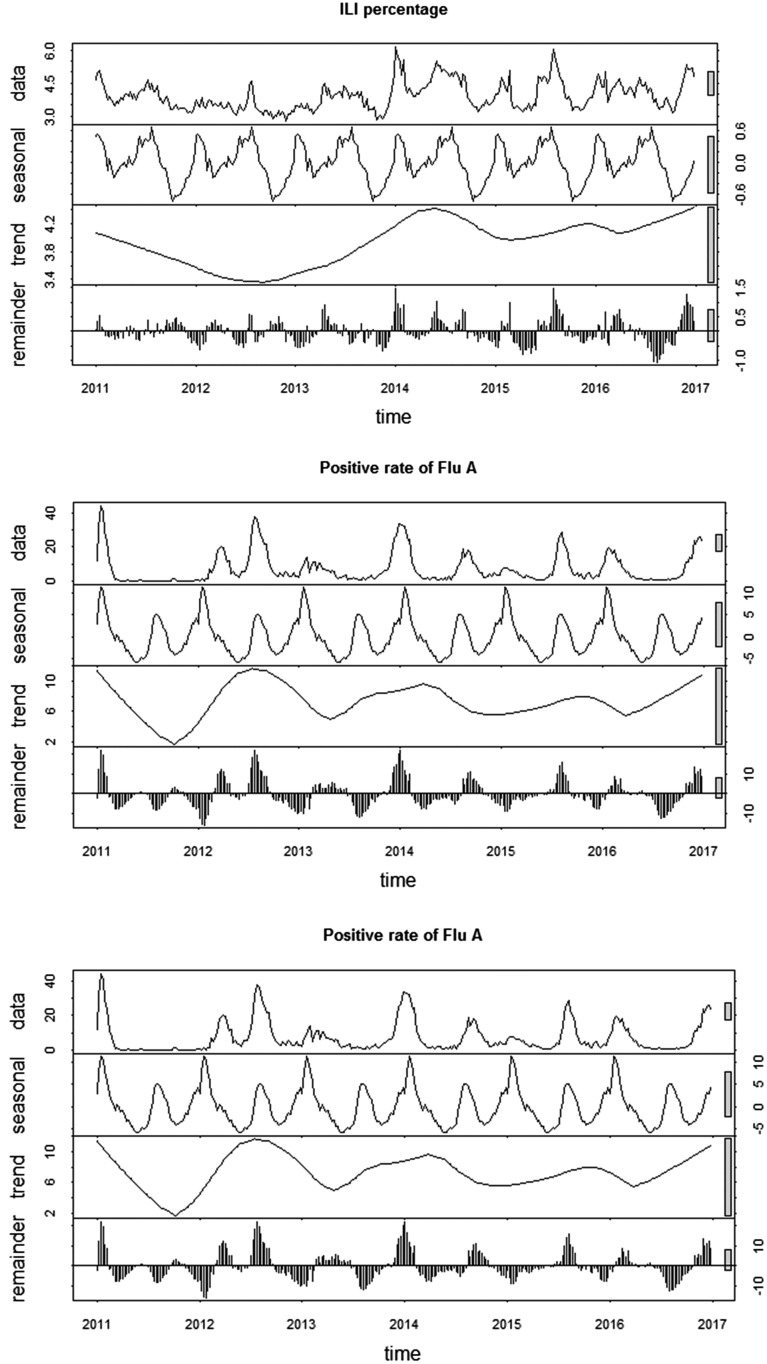
Temporal patterns of ILI activity and influenza virus positive rates in Jiangsu province, 2011–2016.

More than 2 million ILI cases were reported to CISIS from the sentinel sites in Jiangsu province during the study period, with an average weekly ILI of 3.92%. Totally 146 236 throat swabs were sampled from the ILI cases. Influenza viruses were detected in 16 197 swabs through real time RT-PCR, reaching a general positive rate of 11.08%. According to the typing results, Flu-A and Flu-B accounted for 64.27% and 35.73% of all influenza positive samples, reaching an average positive rate of 7.12% and 3.96%, respectively. Two peaks were observed in the ILI activity and the positive rate of Flu-A in each year, one occurred in winter and the other in summer. While the positive rate of Flu-B just showed a winter peak in a year (Fig. 1). The features of the meteorological variables were summarised in Table 1.

**Table 1.** Summary of weekly meteorological variables in Jiangsu province, 2011–2016

Variable	Min	P25	P50	Mena	P75	Max
AP (Pa)	998.6	1006.8	1015.7	1015.2	1022.7	1034.0
Mean_T (°C)	−2.191	6.953	17.092	15.621	23.551	32.648
MAX_T (°C)	1.252	11.622	22.471	20.174	27.467	37.407
MIN_T (°C)	−5.997	3.44	12.698	11.938	20.434	28.199
RH (%)	45.93	67.86	74.40	73.40	80.24	91.18
PR (mm)	0	3.335	11.648	21.296	29.987	159.657
SD (hour)	2.252	27.574	37.274	37.987	48.717	82.009

### Correlation analysis

**Fig. 2.**
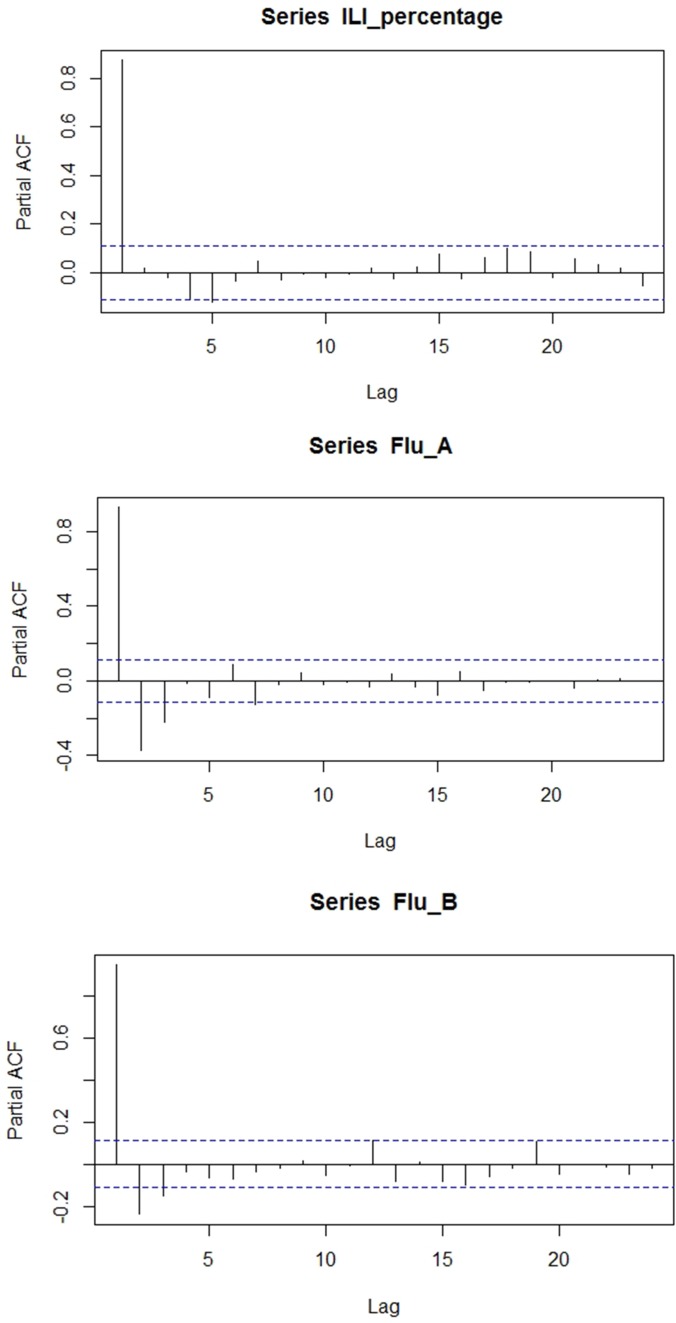
Partial autocorrelation function of time series ILI percentage, positive rate of Flu A and positive rate of Flu B.

As shown in Table 2, AP and PR were significantly correlated with ILI% at lag 0–4. Mean_T and Min_T were also correlated with ILI% but with no lag effect. Max_T, RH and SD presented no relationship with ILI%. As to Flu-A, AP showed correlations at lag 0–3. The three temperature variables presented correlations at lag 0–4. All the meteorological factors were identified significant correlations with Flu-B at lag 0–4. The results of autocorrelation analysis were displayed in Fig. 2. ILI% presented autocorrelation at lag 1, while both Flu-A and Flu-B at lag 3.

**Table 2.** Cross correlation between dependent variable and meteorological factors

Dependent variable	Lag	Correlation coefficient						
		AP	Mean_T	Max_T	Min_T	RH	PR	SD
Weekly ILI**%**	0	−0.195*	0.112*	0.106	0.119*	0.104	0.159*	−0.006
	1	−0.172*	0.081	0.077	0.086	0.067	0.181*	−0.023
	2	−0.146*	0.051	0.047	0.055	0.053	0.153*	−0.061
	3	−0.126*	0.026	0.025	0.028	0.033	0.142*	−0.072
	4	−0.113*	0.006	0.008	0.005	0.033	0.143*	−0.1
Weekly positive rate of Flu-A	0	0.193*	−0.225*	−0.225*	−0.216*	−0.051	−0.059	−0.006
	1	0.181*	−0.221*	−0.222*	−0.210*	−0.06	−0.041	−0.024
	2	0.161*	−0.204*	−0.210*	−0.189*	−0.05	−0.013	−0.051
	3	0.127*	−0.173*	−0.182*	−0.156*	0.002	0.024	−0.103
	4	0.096	−0.139*	−0.152*	−0.120*	0.041	0.038	−0.131
Weekly positive rate of Flu-B	0	0.375*	−0.459*	−0.465*	−0.454*	−0.271*	−0.206*	−0.143*
	1	0.427*	−0.492*	−0.496*	−0.486*	−0.278*	−0.226*	−0.142*
	2	0.461*	−0.516*	−0.519*	−0.510*	−0.291*	−0.236*	−0.144*
	3	0.495*	−0.539*	−0.538*	−0.533*	−0.303*	−0.256*	−0.129*
	4	0.509*	−0.547*	−0.545*	−0.541*	−0.299*	−0.266*	−0.136*

*statistically significant at 0.05.

### RF model fitting and forecasting

Three RF models with optimum parameters were finally constructed to predict ILI activity, Flu-A and Flu-B positive rates in Jiangsu province, including 13, 23 and 39 predictors, respectively. The dependent variable of Flu-A had undergone a natural logarithmic transformation before the model fitting. See Table 3.

**Table 3.** Predictors in different models

Model	lag	ILI-P	Flu-A	Flu-B	time	AP	Mean_T	Max_T	Min_T	RH	PR	SD
RF-ILI_P	0				**√**	**√**	**√**		**√**	**√**	**√**	
	1	**√**				**√**					**√**	
	2					**√**					**√**	
	3					**√**					**√**	
	4					**√**					**√**	
RF-Flu_A	0				**√**	**√**	**√**	**√**	**√**			
	1		**√**			**√**	**√**	**√**	**√**			
	2		**√**			**√**	**√**	**√**	**√**			
	3		**√**			**√**	**√**	**√**	**√**			
	4						**√**	**√**	**√**			
RF-Flu_B	0				**√**	**√**	**√**	**√**	**√**	**√**	**√**	**√**
	1			**√**		**√**	**√**	**√**	**√**	**√**	**√**	**√**
	2			**√**		**√**	**√**	**√**	**√**	**√**	**√**	**√**
	3			**√**		**√**	**√**	**√**	**√**	**√**	**√**	**√**
	4					**√**	**√**	**√**	**√**	**√**	**√**	**√**

**Fig. 3.**
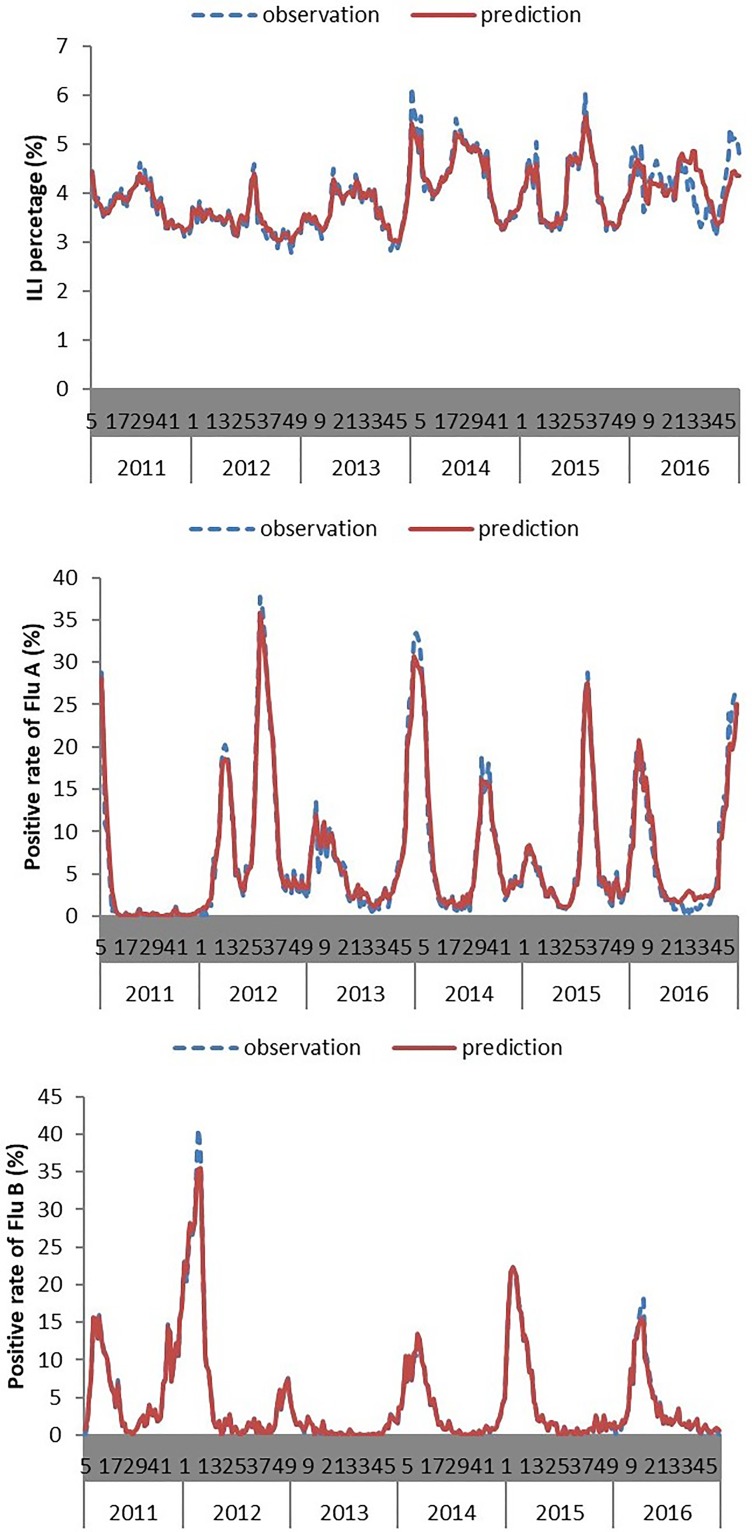
Plot of observed and predicted values via different models.

The performance of the models is summarised in Table 4 and the predicting results are displayed in Fig. 3. The models for Flu-B and ILI% presented excellent performance both in model fitting stage and prospective forecasting stage, with MAPEs less than 10%. The model for Flu-A presented much worse than the other two, with MAPE up to19.49% in the test set. Nevertheless, the predicted values matched the real trend very well.

**Table 4.** Performance evaluation of different random forest models

Model	R^2^		MAPE(%)	
	Train	Test	Train	Test
RF-ILI_P	0.79	0.50	2.48	9.95
RF-Flu_A	0.89	0.0.82	11.24	19.49
RF-Flu_B	0.95	0.80	3.20	8.58

### Variable importance

**Fig. 4.**
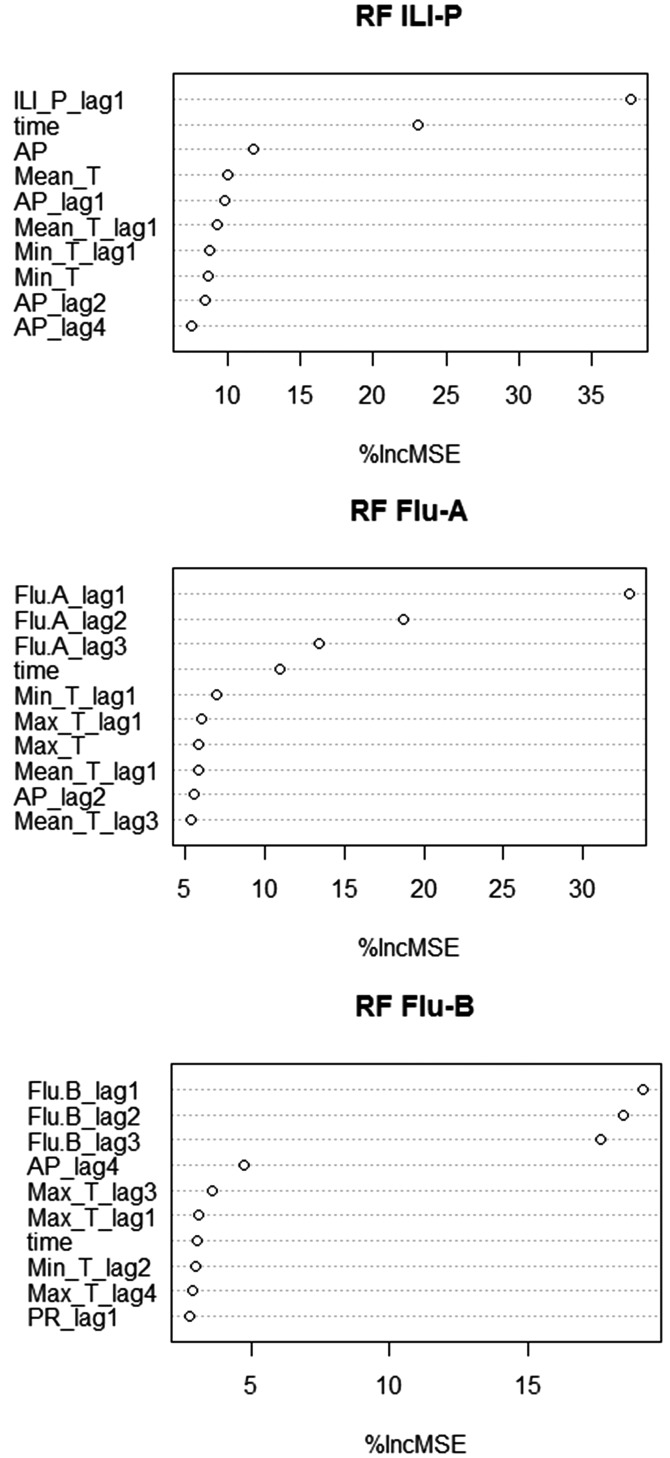
Variable importance in random forest regression models (just displaying the top 10 variables).

In each model, the lagged dependent variable was the most important of all predictors. The time variable presented as important in the models for ILI and Flu-A. Most of the meteorological factors and their lagged terms had the potential to improve the accuracy of the models to a certain degree, but their effects differed across the three models. For ILI forecasting, the weekly MEAN_T, AP and one order lagged AP were more important than the rest. For Flu-A, the lagged temperature specific variables were relatively important. With regard to Flu-B, the lagged AP and MAX_T presented greater effects than the other meteorological variables to improve the model accuracy. See Fig. 4.

## Discussion

Forecasting of influenza activity in human populations is crucial for influenza prevention and control [4]. Many methods have been introduced for this purpose. As a conventional univariate model, ARIMA technique has been commonly used to forecast seasonal influenza surveillance at national, regional and local levels [18–20]. ARIMA model is virtually a linear method. It can achieve good predication when the variation contained in the data is relatively stable. In practice, however, the long-term trend and seasonality of influenza activity change over time, so that the ARIMA model cannot always reach a satisfactory result.

Substantial studies have proposed that influenza activity is climate-sensitive [21–23]. Climatic factors may influence the survival and spread of influenza viruses in the environment, the host susceptibility and exposure probability [24–26]. The effects of meteorological factors on epidemics of ILI have attracted considerable interest recently. Sudarat Chadsuthi, *et al*. [27] fitted ARIMA model with temperature and RH as covariates to forecast the incidence of influenza in Thailand. N'gattia1, *et al*. [28] also developed ARIMA with meteorological variable rainfall to forecast influenza transmission. But the prediction accuracy of these models was not good enough and the climate variables did not clearly optimise the models. In this study, we employed RF algorithm fitting models to predict influenza activity with meteorological factors in Jiangsu province, China. In contrast with previous studies, we constructed predicting models not only for ILI but also for the positive rates of influenza virus (i.e. flu-A and flu-B). All the models performed very well in our dataset. Based on them, we can comprehensively and systematically evaluate the influenza activity in the future, which has significant and practical meaning for influenza prevention and control. Given the good performance of RF in influenza prediction, the models we established could be used for influenza (sub)type-specific early warning and to evoke early intervention. The key meteorological factors identified could be used for publicity, to elevate the general population's consciousness and engagement in influenza prevention.

Similar to many other members of the machine learning family such as artificial neural networks, RF model cannot explain the association between risk factors and influenza activity. But RF can assess the importance of each variable on the accuracy of prediction [14, 29], which is essential to optimise the model and may provide clues for the further study of influenza risk factors. In this study, we found that the lagged dependent variables (i.e. the proportion of ILI in the outpatients and positive rates of flu-A and flu-B) in the previous weeks were more important than meteorological factors in the models. It suggests that these models took advantage of the autocorrelation of the dependent variables. The influenza activity in Jiangsu province presented obvious seasonality which is a critical feature to fit predicting model. However, RF is unable to learn the seasonal patterns because of randomly selecting samples for each tree. In this study, we introduced a time variable into the models to fit the seasonal variance of ILI and positive rates of influenza viruses. The importance analysis shows that it played a significant role to improve the models. This strategy is worthy of reference when fitting the similar RF models. Compared with other multivariate predicting methods [27, 28], RF is not subject to multicollinearity, mainly due to randomly selecting variables for each tree in RF [29, 30]. In this study, we selected predictor variables through cross-correlation analysis. The meteorological factors and their lagged terms were incorporated into the models so long as they were identified to be significantly correlated with the dependent variables. All of them presented some degree of importance, which suggested that the RF models comprehensively combined the climatic variables and their hysteresis effects. Furthermore, the importance of the meteorological factors differed across the three models, which may suggest that the influence of meteorological factors differs between ILI, flu-A and flu-B. The causes of this difference and its practical significance for influenza surveillance deserve further studies.

In this study, humidity and PR were not recognised as major meteorological factors related to ILI activity, positive rate of flu A and B, while the temperature was identified as the main driver. This is consistent with our previous study [31]. The present study also indicates that AP plays an important role in the activity of ILI and flu B. An increased influenza risk associated with rising AP was also reported in another subtropical region in China, using distributed lag nonlinear model [32].

Our study suggests that the selected meteorological variables contributed less to the fluctuations of ILI, flu A and B, compared with the effect of autocorrelation, which has been shown as the most important of independent variables. Monamele GC, *et al*. also supposed that meteorological parameters could only explain no more than 30% of the influenza activity variation [33]. Although our constructed RF models showed desirable predictive ability, especially for ILI and flu B, more meteorological factors, such as specific humidity and absolute humidity, and population-specific immunity level [8] are warranted to be evaluated to improve the prediction of type/subtype-specific activity [34].

## Conclusion

RF model is a good method to predict the influenza activity. Three RF models were constructed to predict the positive rate of influenza viruses and ILI incidence and performed very well. The autocorrelation and seasonal variation contained in the data of the dependent variables are crucial for the prediction models. Meanwhile, the effects of meteorological factors and cumulative effects over a period of time were combined to improve the models. Further researches are warranted to explore RF model with meteorological factors as well as other variables and it has the potential to be a useful tool for predicting other major infectious diseases.
